# Supplementary material for: Derangements and Reversibility of Energy Metabolism in Failing Hearts Resulting from Volume Overload: Transcriptomics and Metabolomics Analyses
Source: Int J Mol Sci. 2022 Jun 18;23(12):6809. doi: 10.3390/ijms23126809 (PMC9224487; doi:10.3390/ijms23126809)
Supplement: Supplementary file 1 [file ijms-23-06809-s001.zip › ijms-1751030-supplementary.pdf]

Table S1. Echocardiography and hemodynamic study

	Sham 8W	CH	CH_{COV}	Sham 16W	HF	HF_{COV}	*p* value: CH vs. Sham 8W	*p* value: CH_{COV} vs. Sham 8W	*p* value: HF vs. Sham 16W	*p* value: HF_{COV} vs. Sham 16W	*p* value: CH vs. HF
FS (%)	47.0 ± 4.0	44.9 ± 3.1	46.0 ± 4.1	47.7 ± 5.6	35.5 ± 5.8	36.2 ± 3.2	0.365	0.704	<0.001	<0.001	0.002
LVEDD (mm)	6.9 ± 0.3	9.4 ± 0.6	8.6 ± 0.9	7.5 ± 0.6	11.4 ± 0.8	8.9 ± 0.5	<0.001	0.005	<0.001	0.0014	<0.001
LVESD (mm)	3.6 ± 0.2	5.2 ± 0.4	4.6 ± 0.7	3.9 ± 0.5	7.3 ± 1.0	5.5 ± 0.5	<0.001	0.02	<0.001	<0.001	<0.001
IVS thickness (mm)	1.7 ± 0.2	2.1 ± 0.3	1.9 ± 0.2	1.7 ± 0.3	2.1 ± 0.3	1.9 ± 0.3	0.022	0.1	0.029	0.241	>0.999
LVEDD/PW thickness	4.1 ± 0.4	4.8 ± 0.4	4.5 ± 0.6	4.0 ± 0.7	5.3 ± 0.6	4.9 ± 0.6	0.013	0.204	0.006	0.038	0.12
LA diameter (mm)	4.0 ± 0.3	6.7 ± 1.0	4.9 ± 0.5	4.8 ± 0.8	6.8 ± 1.3	6.1 ± 0.5	<0.001	<0.001	0.003	0.002	0.922
LVEDP (mmHg)	5 ± 1	7 ± 4	6 ± 3	5 ± 1	12 ± 2	11 ± 3	0.261	0.452	<0.001	<0.001	0.008
+dP/dtmax (mmHg/s)	5684 ± 298	4737 ± 476	5883 ± 433	5899 ± 476	3737 ± 476	3972 ± 452	0.002	0.161	<0.001	<0.001	0.001
-dP/dtmax (mmHg/s)	-5504 ± 324	-4435 ± 438	-4924 ± 542	-5074 ± 432	-3422 ± 358	-3744 ± 432	<0.001	0.039	<0.001	<0.001	<0.001

Abbreviations: CH, compensated hypertrophy; COV, correction of volume overload; +dP/dt, the maximum positive value of the first derivative of left ventricular pressure; -dP/dt, the maximum negative value of the first derivative of left ventricular pressure; FS, fractional shortening; HF, heart failure; IVS, interventricular septum; LA, left atrium; LVEDD, left ventricular end-diastolic diameter; LVEDP, left ventricular end-diastolic pressure; LVESD, left ventricular end-systolic diameter; LVPW, left ventricular posterior wall.

Table S2. Differentially expressed genes (fold change > 1.5 and *p* value < 0.05) involved in glycolysis, the hexosamine biosynthetic pathway, and fatty acid metabolism (Genes involved in the tricarboxylic acid cycle were not significantly changed; data not shown)

Gene symbol	Gene description	Log_2 (fold change)					*p* value				
		CH vs. Sham	CH_{COV} vs. Sham	HF vs. Sham	HF_{COV} vs. Sham	CH vs. HF	CH vs. Sham	CH_{COV} vs. Sham	HF vs. Sham	HF_{COV} vs. Sham	CH vs. HF
**Glucose uptake**											
Slc2a4	solute carrier family 2 (facilitated glucose transporter), member 4	-0.3419	-0.1586	-0.8646*	-0.3559	-0.5228	0.0440	0.1974	0.0070	0.0699	0.0619
**Glycolysis**											
Pfkm	phosphofructokinase, muscle	-0.3427	0.2891	-0.9244*	-0.3785	-0.5816	0.2464	0.3212	0.0047	0.2573	0.0071
Pfkfb1	6-phosphofructo-2-kinase/fructose-2,6-biphosphatase 1	-0.7069*	-0.5204	-0.7464*	-0.6889*	-0.0394	0.0014	0.0408	0.0264	0.0006	0.8802
Aldob	aldolase B, fructose-bisphosphate	-0.4082	-0.2667	-0.7118*	-0.6022*	-0.3036	0.0288	0.1829	0.0203	0.0007	0.2737
Pgam2	phosphoglycerate mutase 2 (muscle)	-0.2426	-0.0736	-0.8518*	-0.3367	-0.6092	0.0472	0.2124	0.0174	0.0132	0.0679
Eno3	enolase 3, beta, muscle	-0.6617	-0.3597	-1.0452*	-0.2330	-0.3836	0.0962	0.0974	0.0048	0.3162	0.2872
**Hexosamine biosynthetic pathway**											
Gfpt2	glutamine-fructose-6-phosphate transaminase 2	0.6040	0.1159	0.7365*	0.2544	0.1325	0.0195	0.6600	0.0451	0.2535	0.6481
Uap1	UDP-N-acteylglucosamine pyrophosphorylase 1	1.0615*	-0.0208	1.0344*	0.1167	-0.0271	0.0011	0.9373	0.0215	0.5112	0.9397
**Fatty acid metabolism**											
Ppargc1b	peroxisome proliferator-activated receptor gamma, coactivator 1 beta	-0.4012	0.1075	-0.6266*	-0.2632	-0.2254	0.0372	0.1608	0.0059	0.0024	0.4842
Rxra	Retinoid X receptor alpha	-0.4429	-0.2918	-0.6918*	-0.3683	-0.2489	0.0078	0.1301	0.0023	0.0024	0.1853
Acsl1	acyl-CoA synthetase long-chain family member 1	-0.2391	-0.0789	-0.6058*	-0.0896	-0.3667	0.1345	0.1907	0.0018	0.2423	0.0638
Acsl6	acyl-CoA synthetase long-chain family member 6	0.1440	0.0290	-0.4446	-0.2660	-0.5886*	0.6103	0.9154	0.1291	0.2713	0.0291
Acsf2	acyl-CoA synthetase family member 2	-0.5694	0.0507	-0.9147*	-0.2779	-0.3453	0.0011	0.8084	0.0047	0.0981	0.1676
Acsf3	acyl-CoA synthetase family member 3	-0.3307	-0.2146	-0.6604*	-0.4038	-0.3296	0.1743	0.1215	0.0094	0.0735	0.2072
Acads	acyl-Coenzyme A dehydrogenase, C-2 to C-3 short chain	-0.3206	-0.1377	-0.8963*	-0.4554	-0.5758	0.0648	0.2723	0.0037	0.0415	0.0307
Acadvl	acyl-Coenzyme A dehydrogenase, very long chain	-0.3718	-0.0614	-0.6529*	-0.2551	-0.2811	0.0038	0.3939	0.0026	0.0177	0.1153
Echdc2	enoyl Coenzyme A hydratase domain containing 2	-0.5552	0.0400	-0.5864*	-0.0096	-0.0312	0.0024	0.7575	0.0027	0.9477	0.8474
Echdc3	enoyl Coenzyme A hydratase domain containing 3	-0.4181	-0.2329	-0.5110	-0.3401	-0.0928	0.0242	0.1646	0.0103	0.0309	0.5188

Ehhadh	enoyl-Coenzyme A, hydratase/3-hydroxyacyl Coenzyme A dehydrogenase	-0.7149*	-0.4026	-0.7903*	-0.5152	-0.0754	0.0207	0.0207	0.0082	0.0153	0.7911
Hadha	hydroxyacyl-Coenzyme A dehydrogenase/3-ketoacyl-Coenzyme A thiolase/enoyl-Coenzyme A hydratase	-0.4056	-0.0500	-0.6540*	-0.2645	-0.2484	0.0014	0.5167	0.0005	0.0411	0.0674
Acaa2	Acetyl-Coenzyme A acyltransferase 2	-0.3567	-0.2701	-0.8397*	-0.7693*	-0.4830	0.2965	0.1952	0.0044	0.0240	0.1682

* denotes $p < 0.05$ compared to the corresponding sham controls.

Table S3. Intermediates of glycolysis

	Sham 8W		CH		CH_{COV}		Sham 16W		HF		HF_{COV}		*p* value						
Name/ppb	Mean	SD	Mean	SD	Mean	SD	Mean	SD	Mean	SD	Mean	SD	CH vs. Sham 8W	CH_{COV} vs. Sham 8W	CH vs. CH_{COV}	HF vs. Sham 16W	HF_{COV} vs. Sham 16W	HF vs. HF_{COV}	CH vs. HF
G6P	78893.1	16650.5	87011.2	15884.3	72153.4	13967.1	74376.9	16394.7	57995.5	28505.4	77834.4	24728.8	0.408	0.465	0.116	0.272	0.801	0.254	0.054
F6P	10853.0	2251.5	11486.5	1906.0	10207.6	1536.7	10918.6	2148.8	7339.0	3924.6	11536.8	3321.3	0.610	0.575	0.230	0.085	0.727	0.091	0.042
FBP	8506.3	1123.5	9358.7	1163.5	8402.9	5364.3	975.9	975.0	8587.9	2201.1	8565.7	1611.6	0.226	0.461	0.708	0.657	0.599	0.985	0.466
G3P	24495.5	4426.8	25658.9	4252.5	22195.5	3929.8	23279.0	4118.7	18901.2	7556.9	24100.4	6275.9	0.652	0.364	0.174	0.269	0.817	0.252	0.085
2PG/3PG	2735.4	451.5	1917.1	487.1	2091.9	661.0	2758.8	409.22	1066.6	293.6	1882.4	568.4	0.013	0.077	0.613	<0.001	0.008	0.013	0.004
PEP	232.1	91.2	161.8	52.8	125.6	29.5	227.7	100.8	112.1	43.2	161.8	45.0	0.133	0.021	0.173	0.030	0.222	0.095	0.105
Pyruvate	92.1	11.7	58.8	27.2	78.7	12.6	97.9	16.3	54.0	5.7	84.9	17.0	0.020	0.087	0.134	0.001	0.226	0.005	0.688

Abbreviations: 2PG, 2-phosphoglycerate; 3PG, 3-phosphoglycerate; CH, compensated hypertrophy; COV, correction of volume overload; F6P, fructose 6-phosphate; FBP, fructose 1,6-bisphosphate; G3P, glyceraldehyde 3-phosphate; HF, heart failure; PEP, phosphoenolpyruvate; SD, standard variation.

Table S4. Intermediates of the hexosamine biosynthetic pathway

	Sham 8W		CH		CH_{COV}		Sham 16W		HF		HF_{COV}		*p* value						
Name/μM	Mean	SD	Mean	SD	Mean	SD	Mean	SD	Mean	SD	Mean	SD	CH vs. Sham 8W	CH_{COV} vs. Sham 8W	CH vs. CH_{COV}	HF vs. Sham 16W	HF_{COV} vs. Sham 16W	HF vs. HF_{COV}	CH vs. HF
Glucosamine-6-phosphate	0.033	0.008	0.033	0.010	0.038	0.011	0.032	0.009	0.040	0.011	0.052	0.015	0.961	0.358	0.416	0.208	0.020	0.198	0.274
UDP	155.9	14.5	210.9	50.4	168.5	42.7	175.7	53.8	170.1	16.0	192.5	38.5	0.044	0.520	0.061	0.817	0.549	0.259	0.119
UDP-N-acetyl-glucosamine	0.282	0.018	0.388	0.048	0.355	0.065	0.309	0.030	0.474	0.055	0.392	0.025	0.002	0.039	0.243	<0.001	<0.001	0.007	0.016

Abbreviations: CH, compensated hypertrophy; COV, correction of volume overload; HF, heart failure; SD, standard variation; UDP, uridine diphosphate.

Table S5. Acylcarnitines

	Sham 8W		CH		CH_{COV}		Sham 16W		HF		HF_{COV}		*p* value						
Name/ppb	Mean	SD	Mean	SD	Mean	SD	Mean	SD	Mean	SD	Mean	SD	CH vs. Sham 8W	CH_{COV} vs. Sham 8W	CH vs. CH_{COV}	HF vs. Sham 16W	HF_{COV} vs. Sham 16W	HF vs. HF_{COV}	CH vs. HF
L-carnitine	55366.7	8215.4	57120.0	1587.3	53266.7	8390.5	58728.0	6505.9	38263.3	13222.2	53443.3	6996.7	0.549	0.638	0.316	0.001	0.148	0.032	0.006
C2 acetyl	14701.8	1589.0	14526.7	2725.7	17565.0	2773.1	14185.7	3655.9	19292.0	2484.2	18342.9	2134.2	0.868	0.081	0.101	0.011	0.012	0.493	0.015
C3 propionyl	259.8	63.0	318.6	47.9	209.7	76.3	248.1	45.2	245.5	46.2	320.2	83.4	0.075	0.188	0.014	0.912	0.024	0.078	0.023
C4 butyryl	245.1	80.5	242.4	54.1	352.9	134.8	207.0	47.0	521.1	88.2	360.9	100.9	0.944	0.082	0.097	<0.001	0.006	0.012	<0.001
C4 isobutyryl	27.1	27.2	31.3	10.7	23.6	16.7	23.5	11.0	45.6	25.1	37.0	16.3	0.719	0.776	0.360	0.014	0.039	0.473	0.227
C5 valeryl	41.9	19.3	48.7	7.3	46.4	23.1	43.7	11.7	65.9	31.1	68.5	22.8	0.285	0.661	0.827	0.029	0.004	0.864	0.240
C6 hexanoyl	51.3	19.4	61.8	18.9	62.0	23.5	46.4	21.7	86.7	22.3	70.8	20.3	0.288	0.316	0.989	0.002	0.030	0.206	0.064
C8 octanoyl	20.5	7.1	26.2	9.7	20.3	3.4	16.5	5.2	29.0	7.9	24.4	8.5	0.186	0.947	0.230	0.002	0.031	0.329	0.586
C10 decanoyl	10.7	3.5	13.0	4.3	14.5	8.6	8.8	2.6	17.6	1.1	13.2	3.6	0.239	0.208	0.716	<0.001	0.010	0.016	0.030
C12 lauroly	13.1	4.4	14.5	4.5	13.6	1.5	12.2	3.8	22.2	4.1	17.7	5.7	0.544	0.767	0.683	<0.001	0.029	0.133	0.011
C14 myristoyl	76.2	37.8	74.7	36.3	97.5	24.0	77.1	29.3	127.2	22.8	154.5	93.4	0.937	0.276	0.260	0.004	0.033	0.502	0.013
C16 palmitoyl	81.0	32.1	104.1	82.6	131.0	9.5	78.9	37.4	169.0	119.3	172.8	134.7	0.417	0.005	0.463	0.034	0.041	0.960	0.314
C18 stearoyl	3.0	0.5	3.2	1.2	3.4	0.2	2.9	0.6	3.9	0.7	3.8	1.3	0.553	0.086	0.777	0.013	0.047	0.931	0.305

Abbreviations: CH, compensated hypertrophy; COV, correction of volume overload; HF, heart failure; SD, standard variation.

Table S6. Intermediates of the tricarboxylic acid cycle

	Sham 8W		CH		CH$_{COV}$		Sham 16W		HF		HF$_{COV}$		*p* value						
Name/ppb	Mean	SD	Mean	SD	Mean	SD	Mean	SD	Mean	SD	Mean	SD	CH vs. Sham 8W	CH$_{COV}$ vs. Sham 8W	CH vs. CH$_{COV}$	HF vs. Sham 16W	HF$_{COV}$ vs. Sham 16W	HF vs. HF$_{COV}$	CH vs. HF
Citrate	48146.7	6388.3	41521.7	10861.3	40253.6	8447.7	41079.7	7145.1	42566.3	14894.7	46832.2	9898.2	0.708	0.824	0.826	0.830	0.275	0.572	0.892
Isocitrate	1996.2	991.5	1124.7	350.3	1288.4	488.3	1546.3	556.9	1980.4	1758.5	1470.2	561.4	0.070	0.362	0.520	0.585	0.818	0.514	0.270
Succinate	576291.7	314916.6	1480521.5	908208.2	1060302.4	438220.4	1082788.2	682330.4	1401680.5	896304.2	1247481.6	910422.0	0.129	0.551	0.339	0.504	0.730	0.774	0.888
Fumarate	51768.1	36331.9	82106.8	58713.9	81058.0	71994.9	60637.1	28664.3	109616.5	61868.9	91408.4	48602.7	0.639	0.692	0.978	0.116	0.211	0.597	0.469
Malate	84468.4	42376.9	84150.8	39524.2	106978.7	42063.0	63426.2	31751.3	77899.4	21141.3	122671.4	79132.1	0.535	0.075	0.378	0.375	0.120	0.429	0.740

Abbreviations: CH, compensated hypertrophy; COV, correction of volume overload; HF, heart failure; SD, standard variation.

Table S7. Oligonucleotide sequences of primers

Pdp2	Forward	5'-GAGGATACTAGGCTGAAAAAC-3'
	Reverse	5'-CCTCCATTTCACTTGATTCC-3'
Ehhadh	Forward	5'-GTTGCTCTTGACCTAATTACC-3'
	Reverse	5'-GCAAATTTGATGGCTTCTTC-3'
Acaa2	Forward	5'-CTACTTTGTGTCTGGATGTG-3'
	Reverse	5'-ATGCTTCATTCACGTCTATC-3'
Acads	Forward	5'-TCCAAAACATCCAGTTCAAG-3'
	Reverse	5'-GGTGAAAGGCTTCTTATTGTC-3'
Pfkfb1	Forward	5'-GGCTATAAGACCTTCTTTGTTG-3'
	Reverse	5'-TTCTTCATCCAGAGACTCATAG-3'
Eno3	Forward	5'-CCAAAGAAGACATTACAGCC-3'
	Reverse	5'-CAGGTATCGTGATTTGTCTC-3'
Rxra	Forward	5'-TCTTCAACCCTGACTCTAAG-3'
	Reverse	5'-CTAGTGATGCATACACCTTC-3'
Aldob	Forward	5'-CATCATGTTTACCTTGAGGG-3'
	Reverse	5'-GACAAAAAGCAGATACCAGG-3'

Supplemental figures

A. Canonical pathway analysis in the CH phase

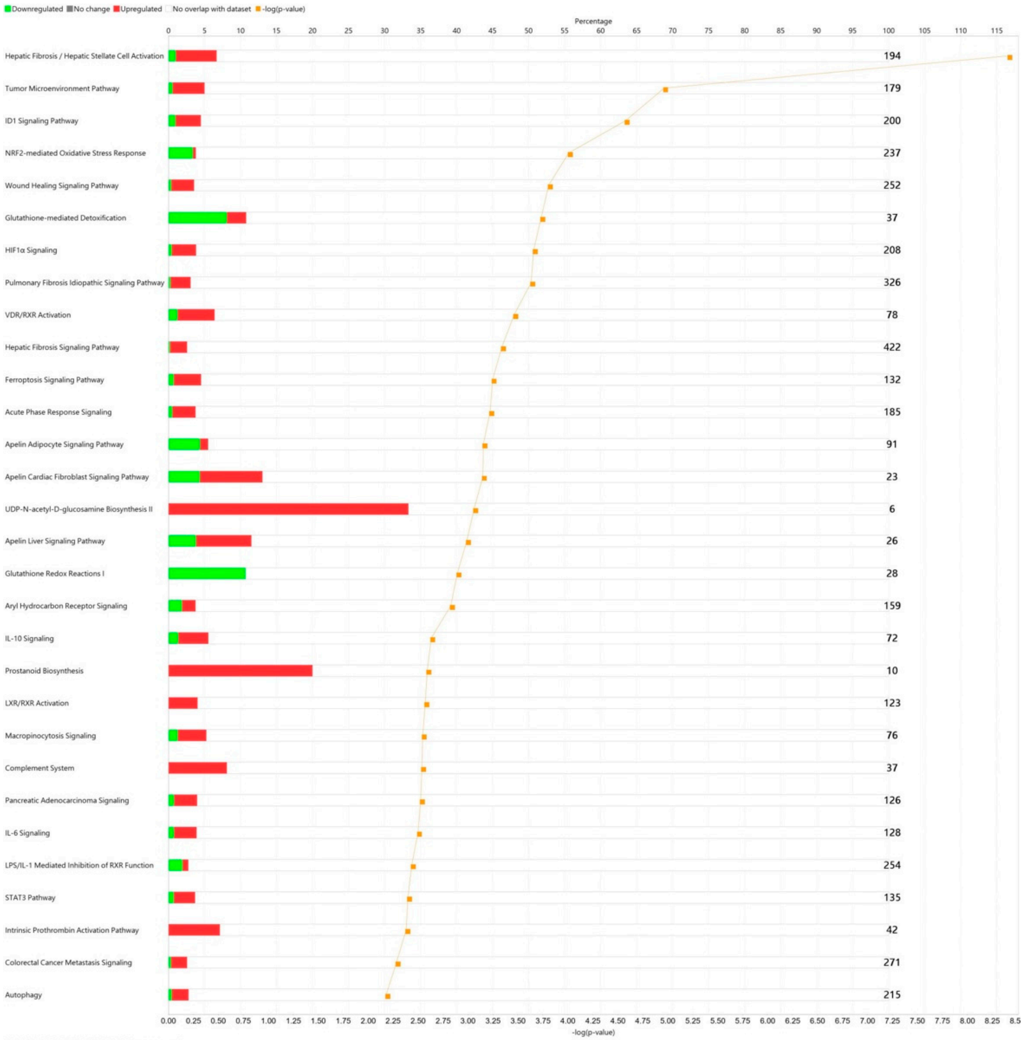

© 2000-2022 QIAGEN. All rights reserved.

B. Canonical pathway analysis in the CH_{COV} phase

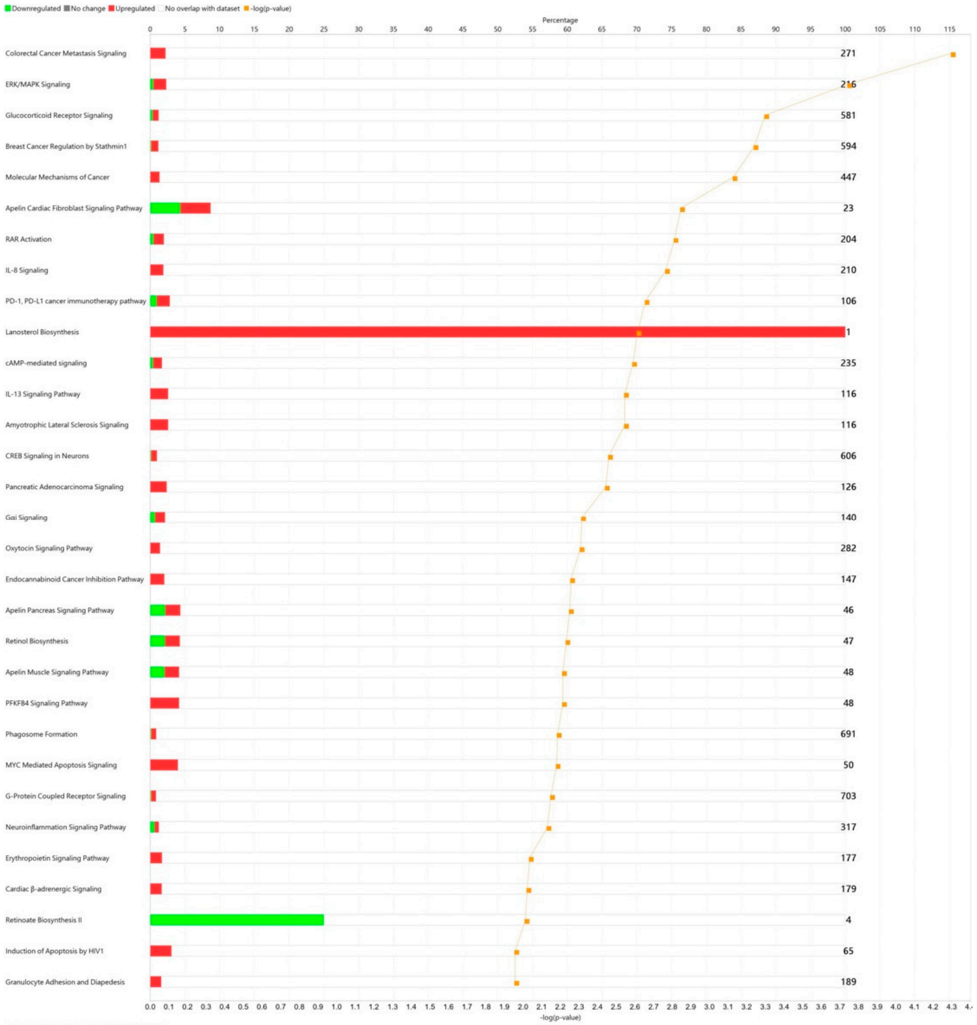

© 2000-2022 QIAGEN. All rights reserved.

Figure S1. Transcriptomics analyses of rats with volume overload (VO) or with correction of VO. Pathway enrichment analyses of the differentially expressed genes (fold change > 1.5 and P < 0.05; ranked by -log [P value] of each pathway) in volume-overloaded rats in the compensated hypertrophy (CH) phase (A) and in VOed rats with correction of VO in the CH phase (CH_{COV}). n = 4 per group.

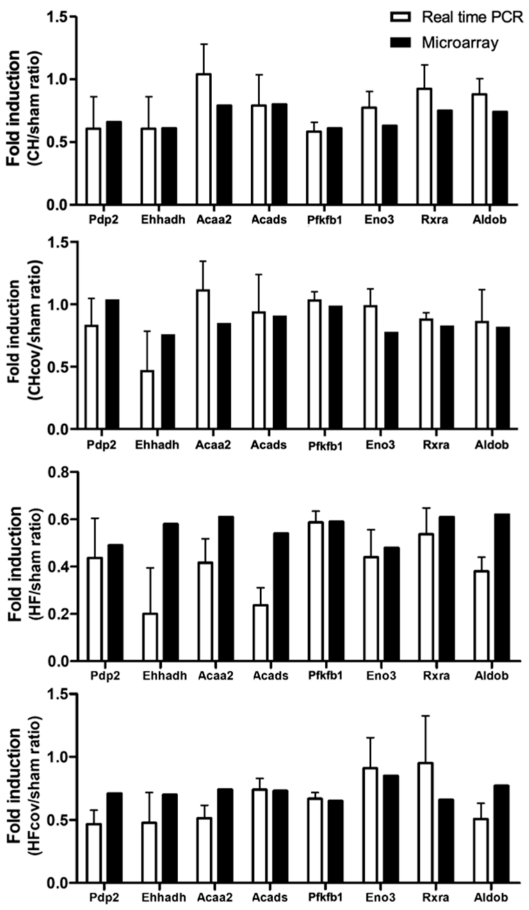

Figure S2. Comparison between quantitative polymerase chain reaction (qPCR) and microarray analysis of specific metabolic genes in different phases of volume overload. Data are expressed as log fold change. Genes with downregulation in the microarray analysis in at least one phase investigated were selected. n = 4 per group. CH, compensated hypertrophy, CH_{COV}, correction of volume overload in the compensated hypertrophy phase; HF, heart failure; HF_{COV}, correction of volume overload in the heart failure phase.

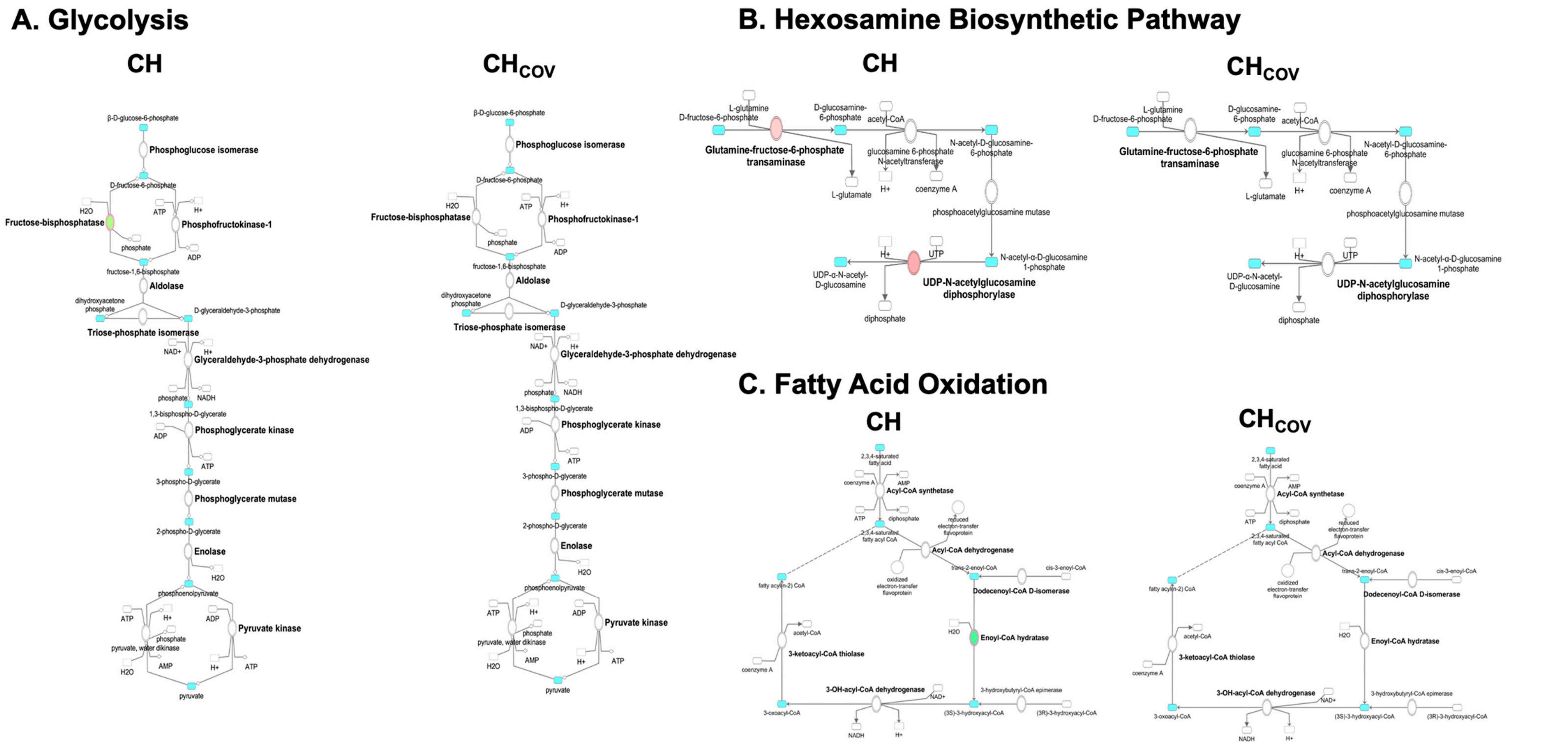

Figure S3. Differential expression of genes (fold change > 1.5 and P < 0.05) involved in glycolysis (A), hexosamine biosynthetic pathway (B), and fatty acid oxidation (C) in volume-overloaded rats in the compensated hypertrophic (CH) phase and in volume-overloaded rats after correction of volume overload in the CH phase (CH_{COV}). The ovals in the maps represent enzymes in each metabolic pathway, with red and green denoting upregulation and downregulation of gene expression, respectively. n = 4 per group.
